# Reproducibility of Intra- and Inter-scanner Measurements of Liver Fat Using Complex Confounder-corrected Chemical Shift Encoded MRI at 3.0 Tesla

**DOI:** 10.1038/srep19339

**Published:** 2016-01-14

**Authors:** Bing Wu, Wei Han, Zhenhong Li, Yonghua Zhao, Mingmei Ge, Xueqing Guo, Xinhuai Wu

**Affiliations:** 1 Radiology Department, Beijing Military General Hospital, Beijing, China; 2 Radiology Department, PLA 263 Hospital, Beijing, China; 3 Department of Clinical Laboratory, PLA 263 Hospital, Beijing, China

## Abstract

The purpose of this study was to prospectively evaluate the reproducibility of the proton density fat-fraction (PDFF) of the liver using the IDEAL algorithm, a quantitative confounder-corrected chemical-shift-encoded MRI method. Data were obtained from 15 volunteers on four different days. The first and the third examinations were conducted on scanner one with one-week intervals, while the second and the fourth tests were performed on scanner two with same time interval. For each test, two MR scans were performed, one before and one after a meal. Regions-of-interest measurements were manually calculated to estimate the PDFF in the right and left lobes on the PDFF images. Reproducibility was measured using the intra-class correlation coefficient (ICC). The ICCs of the PDFF in the right and left lobes were 0.935 and 0.878, respectively. The intra-scanner ICCs of the right lobe before and after a meal or at a one-week interval were 0.924 and 0.953, respectively. The inter-scanner ICCs of PDFF the next day and at a one-week interval were 0.920 and 0.864, respectively. The PDFF of liver derived from IDEAL demonstrated high intra- and inter-scanner measurement reproducibility. The PDFF of the right lobe before a meal was more reproducible than after-meal measurements.

Hepatic steatosis is characterized by the excessive accumulation of lipids within hepatocytes, which is an essential feature of diffuse liver diseases, such as nonalcoholic fatty liver disease, alcoholic liver disease, viral hepatitis, genetic lipodystrophies, cystic fibrosis liver disease, and hepatotoxicity due to numerous therapeutic drugs. Liver biopsy, the current clinical gold standard for the assessment of liver fat, is invasive and produces sampling errors, which makes it a suboptimal tool to diagnose and characterize the severity of steatosis or to participate in the longitudinal follow-up of patients with liver fat.

Magnetic resonance (MR) approaches can break down the liver signal into the corresponding fat and water signal components and provide more direct measurements of liver fat than those of ultrasound (US) or computed tomography (CT). Advanced MR applications measure the proton density fat-fraction (PDFF), defined as the proportion of the mobile proton density in liver tissue attributable to fat, which is a fundamental tissue property and represents the direct measurement of liver fat content. These advanced MR approaches present accurate fat quantification with a commercially available cost. A recently described complex confounder-corrected chemical shift encoded the fat-water separation method, which is based on IDEAL (Iterative Decomposition of water and fat with Echo Asymmetry and Least squares estimation), has been described for fat quantification in the liver^1^,^2^,^3^,^4^,^5^,^6^,^7^.

A clinically applicable fat quantification technique must be both accurate and robust. The reproducibility of one technology refers to the proximity in agreement between a series of measurements obtained from the same subject scanned under different conditions. Therefore, the reproducibility of the fat-fraction technique is essential to ensure that the quantified data can be pooled from different time scales or from different scanners. The current prospective pilot study was conducted to assess the reproducibility of PDFF as a biomarker for liver fat content *in vivo*, which aimed to evaluate the reproducibility of different scanners at different time scales, in different lobes of the liver, or under different levels of stomach emptiness on two respective 3.0 T MR scanners with the same algorithm, IDEAL IQ (software product, GE Healthcare).

## Results

**Figure 1:**
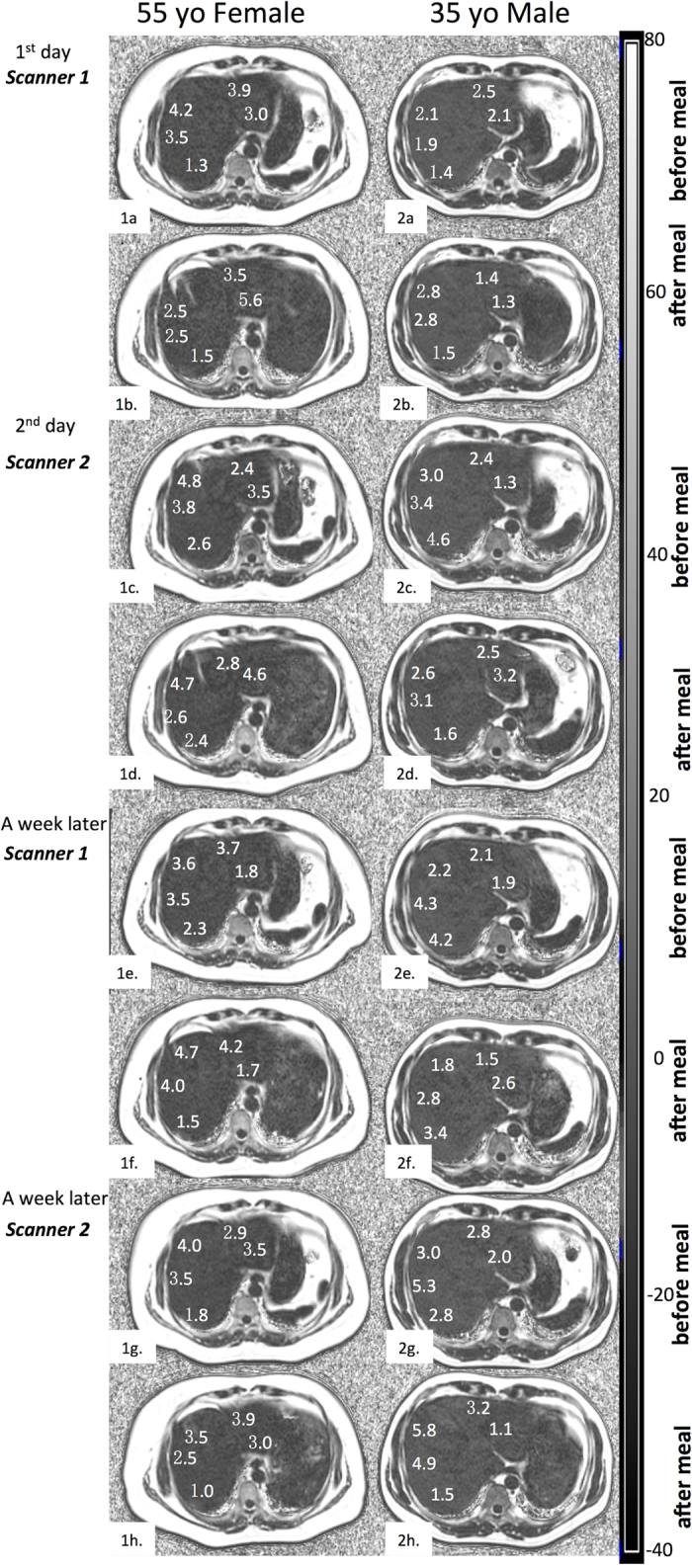
Representative transverse PDFF imaging (in units of %) obtained from two volunteers during eight tests on four respective days. PDFF maps were set with window level of 20 and window wide of 120. MRI-determined PDFF parametric maps of two subjects (55-year-old female, from 1a. to 1 h; 35-year-old male, from 2a to 2 h), demonstrating direct comparison of liver fat content for five ROIs placed on each liver during eight tests on four days. The close qualitative and quantitative agreements in MRI-determined PDFF on two 3.0-T scanners were recorded. The parametric maps were generated from source images by applying the pixel-by-pixel PDFF modelling algorithm of the commercial version. The parametric maps are displayed with a PDFF ranging from 0%–100%. Subcutaneous adipose tissue appeared to be white on the parametric maps because the fat fraction in the adipose tissue was approximately 100% PDFF.

Hepatic PDFF ranged from 0.39%–9.29% (mean: 3.35% +/− 1.96%). Twelve subjects (12/15) exhibited 0%–5% PDFF, while the remaining three exhibited 6%–10% (Fig. 1).

The ICCs of PDFFs before and after meals in the right and left liver lobes across all tests were 0.935 and 0.878, respectively. The intra-scanner ICC of the right liver lobe before and after meals was 0.924. The intra-scanner ICC of the right liver lobe before meals at one-week intervals was 0.953. The inter-scanner ICCs of the PDFF performed the next day and after one week were 0.920 and 0.864, respectively (Table 1).

**Figure 2:**
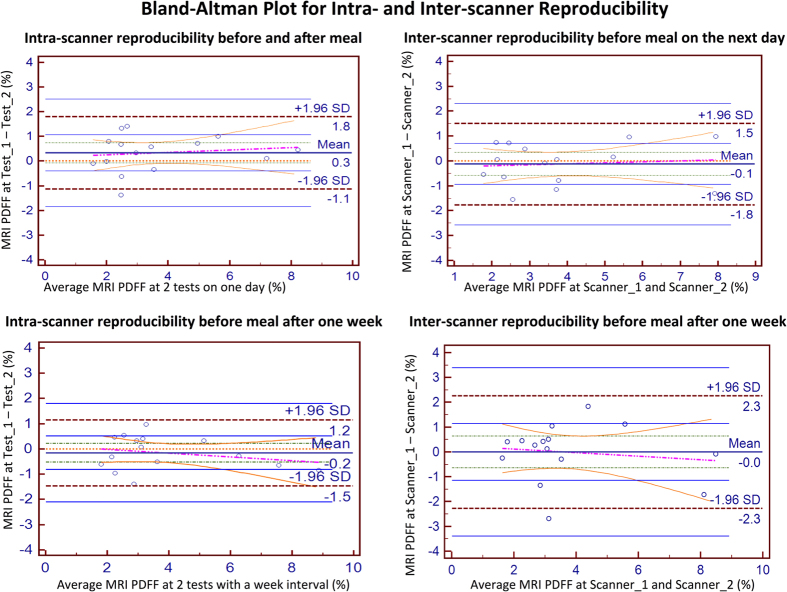
Bland–Altman plots of MRI-determined PDFF estimates on two 3.0-T scanners. The Bland–Altman plots demonstrated a larger measurement error before and after meals on two consecutive days (top left plot) than that of the right liver lobe before meal with one-week interval (bottom left plot). Therefore, food consumption could affect the reproducibility of PDFF measurements. Among the inter-scanner changes of the PDFF, the 95% limits of agreement were smaller for PDFF on the next day (top right plot) than after one week (bottom right plot), indicating that reproducibility decreased as the time increased.

The B-A analysis’ bias (limits of agreement) of the right and left liver lobe was 0.3% (−1.1%–1.7%) and −0.1% (−1.9%–1.6%), respectively. The intra-scanner B-A of the right liver lobe before and after meals was 0.3% (−1.1%–1.8%). The intra-scanner B-A of right liver lobe at a one-week interval was −0.2% (−1.5%–1.2%). The inter-scanner B-As of the PDFF the next day and after one week were −0.1% (−1.8%–1.5%) and 0% (−2.3%–2.3%), respectively (Fig. 2).

Bland–Altman analysis revealed that the PDFF of the right liver lobe was closer than that of the left liver lobe. The B-A results and the bias (limits of agreement) of the right and left liver lobes were 0.3% (−1.1%−1.7%) and −0.1% (−1.9%−1.6%), respectively. The intra-scanner B-A of the right liver lobe before and after meals demonstrated a measurement error of (−1.1%−1.8%), which was larger than that of the right liver lobe before meals after one week (−1.5%−1.2%). Concerning the inter-scanner B-As of PDFF, the 95% limit of agreement was smaller for the PDFFs the next day (−1.8%−1.5%) than after one week (−2.3%−2.3%), which indicated that the reproducibility was slightly reduced as the interval increased (Fig. 2).

## Discussion

There were three major findings in the current study. First, the IDEAL IQ sequence provided PDFF measurements with high intra- and inter-scanner reproducibility in same type of 3.0-T MR scanners in healthy or mild liver steatosis subjects. Secondly, the right lobe of the liver demonstrated better reproducibility for PDFF, while the left lobe possessed acceptable reproducibility. Thirdly, food consumption might affect the reproducibility of the PDFF measurements. To the best of our knowledge, this is the first study to assess the effects of meals and the type of liver lobe on the reproducibility of MRI-based PDFF on two identical version 3.0 T MR scanners. The influence of the detected factors on intra- or inter-scanner reproducibility was significant for the validation of MRI PDFF to function as a biomarker of liver fat content.

PDFF is a promising non-invasive MRI technique to measure regional liver PDFF *in vivo*. In clinical practice, any potential error in measurement may decrease the accuracy of the diagnosis, and clinicians must assess whether the observed changes reflect the actual clinical status of a patient. To evaluate the feasibility of PDFF as a biomarker for clinical application, it is essential to investigate the reproducibility. Preliminary studies have demonstrated the accuracy of MRI-determined PDFF against spectroscopy and its within-examination repeatability^8^,^9^,^10^,^11^, but the majority of the studies concentrated on a single 1.5 T or 3 T MR scanner. However, few studies have investigated the reproducibility of PDFF between different MR scanners. Kang GH *et al*.^12^ observed a high correlation between PDFF measurements using two scanners of different field strengths (1.5 T and 3 T, respectively), where the observed MRI-determined PDFF differences at 1.5 T compared to 3 T ranged from −3.2 to +4.6%. The observed limits of agreement in Kang’s study were −1.9 and +3.7% on the same day, which almost doubled the observed results in the current study (−1.8 and +1.5% on two 3 T scanners on two consecutive days). Secondly, the inter-scanner agreement in the current study was better than that in Kang *et al*.’s study; the 1.5 T estimates were higher by 0.92% on average. In the current study, the bias of PDFF from two scanners on two consecutive days and at one-week intervals were −0.1% and 0%, respectively. Moreover, the imaging range in this study was different from that of Kang *et al*.’s study. In the current study, the PDFF maps were displayed with a range of 0–100% (Fig. 1) versus 0–50% in the previous study, which better mimicked a real clinical situation.

A secondary purpose of the present study was to assess the reproducibility of PDFF on the left and right lobes. As in the prior study^9^, close agreement between imaging and spectroscopy in all sampled segments suggested the high accuracy of imaging over the entire liver. However, in addition to the accuracy of measurements, reproducibility is regarded as another important concern in longitudinal studies. The observed limits of agreement of the right and left lobes were −1.1% to 1.7% and −1.9% to 1.6%, respectively, while the biases of the right and left lobes were 0.3% and −0.1%, respectively. Therefore, the right lobe of liver demonstrated better reproducibility than the left lobe.

Another interesting finding in this study was that food consumption affects the reproducibility of liver PDFF measurements. The limits of agreement in the right liver lobe before and after meals detected on the same scanner were −1.1% and 1.8%, respectively, and the limits of agreement in the right lobe before meals at a one-week interval on same scanner were −1.5% and 1.2% with a bias of 0.3% and −0.2%, respectively. Therefore, the intra-scanner reproducibility of PDFF measurements before and after meals was superior to that of the measurements before meals at a one-week interval.

In the current study, a commercial pulse sequence was applied with a low flip angle of 3 degrees, which was performed in multi-centre longitudinal studies. A previous study showed that a flip angle of 2 or 3 degrees provided the highest accuracy, while PDFF was reproducible with flip angles of 1–5 degrees^11^. The one-day and one-week intervals were selected and investigated in our study, as it was hypothesized that the PDFF of the liver would change very little within these intervals.

Several limitations exist in the current study. First, severe liver steatosis patients could not be included, as a four-day scanning period was too overwhelming for most patients, especially when two MR scanners were located at two hospitals. However, the exclusion of severe patients provided advantages, including relatively good compliance and decreased disease-related variability. Secondly, this study was performed on a small subset of healthy volunteers and mild steatosis cases, and all of them are young or middle-aged. Further studies with a larger sample of both severe steatosis patients and elderly individuals should be conducted. Another limitation of this study was the evaluation of diurnal variation. It would be valuable to know whether there are any changes in PDFF in the liver at different times of the day. Finally, in the absence of a reference standard for liver steatosis measurements, systematic errors were not evaluated.

In conclusion, the PDFF of the liver consistently derived from IDEAL IQ demonstrated high intra- and inter-scanner measurement reproducibility. The PDFF of the right liver lobe before meals was more reproducible for the quantitative estimation of liver fat, which may be applicable in clinical practice.

## Methods

### Subjects and MR Acquisition

Inclusion criteria included healthy adult volunteers or adults with risk factors for NAFLD (overweight, obese, or family history of NAFLD), while exclusion criteria included contraindications to MRI or pregnancy. All subjects provided written informed consent prior to the scan and the study was approved by the Ethics Committee of the Beijing Military General Hospital. All methods were carried out in accordance with the approved guidelines.

The details of each subject were recorded, including age, biological sex, body mass index (BMI), and hepatic function panel results. Eventually, fifteen healthy volunteers (7 males, 8 females) were recruited, with age ranging from 22 to 57 years and a mean of 33.1 years. BMI ranged from 17.9 to 31.6 kg/m^2^. Five volunteers were overweight (body mass index ≥25 kg/m^2^), and 10 (healthy volunteers) were of normal weight.

All the participants were scanned eight times on two scanners on four respective days. The two scanners were located at two different research centres. The first and the second tests were performed on two consecutive days, whereas the third and the fourth were performed on another two continuous days. The first and third tests were conducted on scanner one, while the second and fourth tests were performed on scanner two, both with one-week intervals. Randomization was designed to perform each exam on all the subjects.

Subjects underwent examinations in the supine position with a standard eight-channel torso phased-array coil centred over the liver on 3 T MR system (HDx Signa MR 750 system, GE Healthcare, Waukesha, WI, USA). MR images of the liver were obtained by performing a commercial version of a chemical shift-based water-fat separation method known as IDEAL IQ, implemented with a multi-echo 3D spoiled gradient-echo acquisition to achieve six echoes with “fly-back” gradients. Both scanners were the same version of the GE Signa scanner. During each scan, water–fat separation was performed by using complex-based (T2*-IDEAL), magnitude-based, and the hybrid methods on the same source of data. PDFF images were generated from the separated water and fat images and were evaluated for each assessment.

To minimize T1 effects, a low flip angle of 3° was introduced to reduce T1 bias, and a parallel imaging method, namely ARC (Auto-calibrating Reconstruction for Cartesian acquisition, GE Healthcare) was performed with an acceleration factor of 2 to obtain a sufficient coverage while keeping the scan time within a single breath hold (approximately 21 s). To avoid water/fat swaps, a region-growing algorithm was employed for B0 estimation in the image reconstruction. The remaining parameters of IDEAL IQ were as follows: TR/TE1/ΔTE = 7.2/1.3/0.7 ms; receiver bandwidth = ±166.7 kHz; FOV = 26–33 cm; matrix = 160 × 160; slice thickness = 10 mm.

### Data Processing

PDFF maps were auto-generated on the host scanner after each acquisition of IDEAL IQ. All the images were independently evaluated on the AW4.5 workstation (Advantage Workstation 4.5; GE Healthcare). To estimate the PDFF, one reviewer (with 8-year experience) blinded to the clinical records manually performed circular region-of-interest (ROI) measurements in the right and left liver lobes using OsiriX software on a MacOS X operating system. The PDFF images are shown in Fig. 1. ROIs were selected to be ≈400 mm^2^ in size in the right lobe and ≈300 mm^2^ in size in the left lobe, avoiding visible blood vessels and artefacts. For each series, 9 ROI measurements (three ROIs per slice × 3 levels) were selected in the right lobe, while 4 ROI measurements (two ROIs per slice × 2 levels) were selected in the left lobe. The mean values of the 9 ROIs in the right lobe and 4 ROIs in the left lobe were averaged separately for each corresponding series.

### Statistical Analysis

Reproducibility was measured using the intra-class correlation coefficient (ICC)^13^ and Bland–Altman (B-A) analysis. ICC measures the contribution of inter-subject variances to total variance, which presents the ability of a method to detect the differences between subjects consistently. ICC values range from 0–1, and a value close to 1 indicates high reproducibility. In the current study, one-way random effects model single-measure ICC values were calculated using SPSS (SPSS Inc. v18.0, Chicago, IL). A p-value < 0.05 was regarded as the threshold of statistical significance.

The reproducibility was also assessed by calculating a Bland-Altman plot, which was generated to illustrate the agreement between the PDFF estimates derived from different times on the two scanners. The 95% limits-of agreement and the bias between two PDFF measurement values were obtained as well.

## Additional Information

**How to cite this article**: Wu, B. *et al*. Reproducibility of Intra- and Inter-scanner Measurements of Liver Fat Using Complex Confounder-corrected Chemical Shift Encoded MRI at 3.0 Tesla. *Sci. Rep*. **6**, 19339; doi: 10.1038/srep19339 (2016).

## Figures and Tables

**Table 1:** The ICCs of the right and left liver lobes for intra- and inter-scanner proton density fat-fraction (PDFF) imaging.

		ICC	95% CONFIDENCE INTERVAL	
TOTAL	Right lobe ICC before and after meal	0.935	0.892	0.961
	Left lobe ICC before and after meal	0.878	0.801	0.925
RIGHT LOBE	Intra-scanner ICC before and after meal	0.924	0.795	0.974
	Inter-scanner ICC before meal on the next day	0.920	0.784	0.972
	Intra-scanner ICC before meal after one week	0.953	0.869	0.984
	Inter-scanner ICC before meal after one week	0.864	0.652	0.952
LEFT LOBE	Intra-scanner ICC before and after meal	0.833	0.584	0.940
	Inter-scanner ICC before meal on the next day	0.905	0.749	0.967
	Intra-scanner ICC before meal after one week	0.904	0.745	0.966
	Inter-scanner ICC before meal after one week	0.912	0.755	0.970
